# Analysis of the effect of tibial torsion on tibial osteotomy in knee arthroplasty using a three-dimensional computed tomography-based modelling technique

**DOI:** 10.1186/s12891-019-2744-4

**Published:** 2019-08-07

**Authors:** Yeran Li, Yu-Hang Gao, Lu-Ding, Jianguo Liu, Chen Yang, Ming Li, Xin Qi

**Affiliations:** Department of Orthopaedic Surgery, The First Hospital of Jilin University, Xinmin St 71, Changchun, 130021 Jilin China

**Keywords:** Tibial torsion, Alignment deviation, Knee arthroplasty, Extramedullary system, 3D model

## Abstract

**Background:**

Extramedullary systems are commonly used in knee arthroplasty, with the rod location being determined from the tibial torsion line during surgery. The traditional method for tibial torsion measurement is not in accordance with clinical practice. This study aimed to evaluate proximal and distal tibial torsion using 3-dimensional (3D) computed technology to establish a new evaluation method, as well as to investigate the association between tibial torsion and postoperative alignment deviation.

**Methods:**

Fifty-five osteoarthritis tibias with >10°varus preoperatively were divided into valgus, neutral, and varus groups based on their postoperative alignment deviation. A new method based on clinical practice was built using a 3D tibial model. Proximal and distal tibial torsions were measured by both the new and traditional methods. In addition, tibial osteotomy that followed the intramedullary osteotomy system was simulated on the 3D model in the varus and valgus groups to investigate the association between tibial torsion and alignment deviation.

**Results:**

Proximal tibial torsion was smaller and distal torsion was greater in the valgus group than the other two groups, according to the new method (*p* = 0.03 and *p* = 0.02, respectively). No significant difference was found when comparing these torsions by the traditional method (*p* = 0.782 and *p* = 0.753, respectively). In the valgus group, the postoperative alignment deviation improved after simulated osteotomy guided by the intramedullary system, while no significant improvement was found in the varus group.

**Conclusion:**

According to this new tibial-rotation evaluation method, valgus deviation in knee arthroplasty was identified as the main cause for knees in which the proximal tibial internal torsion is too small and the distal external torsion is too great. The use of an intramedullary system may help reduce this deviation.

**Trial registration:**

Prospectively registered.

## Background

In total knee arthroplasty (TKA), establishing precise alignment is crucial for postoperative function and the durability of the prosthesis [1–3]. For tibial osteotomy, the extramedullary system is intraoperatively used to establish tibial alignment and determine the angle for the osteotomy. The proximal location of the extramedullary rod is determined by the tibial torsion line, which is drawn from the medial one-third of the tibial tubercle to the posterior cruciate ligament during surgery [4–7]. So, the tibial component rotational alignment is not only influenced tibial component’s axial rotation position, but may also influenced tibial component’s coronal alignment. Previously, Dalury et al. suggested that using the internal one-third of the tibial tubercle in the proximal tibial position as an extramedullary locator during TKA could achieve satisfactory results with respect to tibial alignments in 92% of cases [4]. However, Howell et al. showed that the proximal position of the tibia extramedullary locator placed on the medial or medial one-third of the tibial tubercle resulted in tibial prosthesis misalignment and poor internal rotation in 30 and 14% of patients, respectively [5]. Despite the strict use of extramedullary systems during surgery, there still remain some patients with poor tibial alignment. Current studies of tibial torsion generally use the transverse diameter of the tibial plateau as the transtibial axis, which we called as traditional method [8–10]. However, this method is not in accordance with clinical practice: the line that connects the medial one-third of the tibial tubercle and the posterior cruciate ligament are used to determine proximal tibial torsion and to confirm proximal position of the extramedullary rod, instead of the transtibial axis, during surgery. Therefore, the traditional method used in previous studies is not suitable for evaluating the effect of tibial torsion on alignment deviations.

This study aimed to evaluate proximal and distal tibial torsion using a new 3-dimensional (3D) computed technology method which is more reflective of clinical scenarios. To investigate the association between tibial torsion and postoperative alignment deviation, and to compare the difference of alignment deviation between extramedullary and intramedullary simulated osteotomy.

## Methods

### Data collection

We prospectively enrolled patients who received primary knee arthroplasty at our department between June 2017 and January 2018. Patients who had a history of knee trauma or surgery, as well as those with preoperative rheumatoid arthritis, infection, or cancer were excluded from the study. Medical history and radiographic findings were collected. A total of 55 patients diagnosed osteoarthritis with 10^o^ tibio-femoral varus deformity on the preoperative whole leg standing anteroposterior (AP) view were recruited, of which 5 were male and 50 were female. Patient ages ranged from 50 to 88 years and all patients had Kellgren-Lawrence (KL) grade IV knees. Preoperatively, full-length computed tomography (CT) imaging (GE Discovery CT750 HD scanner, GE Healthcare, Waukesha, WI, USA), using a 1.5-mm slice thickness, was conducted on all patients.

An experienced senior orthopaedic surgeon performed all surgeries. All instruments, extramedullary systems, and knee prostheses used in the study were obtained from the same company (PFC Sigma, Depuy Synthes, Warsaw, IN, USA). In addition, long-leg weight-bearing radiography was performed preoperatively to evaluate alignment deformities, as well as postoperatively to examine the alignment in the coronal position. Coronal alignment deviations on full-length postoperative radiography was evaluated by two other senior orthopaedic surgeons who had no knowledge of patient identity or preoperative imaging results. Regarding postoperative alignment, the valgus angle of the postoperative alignment was recorded as a negative value and the varus angle was indicated by a positive value. Malalignment was defined on the strict basis of mechanical alignment, therefore, 0° was neutral, <0°represented valgus and >0°represented varus. This study was approved by the ethics committee of our hospital. All patients were fully informed of the details of study participation and provided informed consent. Among the 55 cases, 19 patients showed valgus deviations (0–5°), while 19 showed varus deviations (0–5°) after surgery; the remaining 17 patients had neutral alignment. Therefore, the patients were divided into three groups: varus, valgus, and neutral groups.

### New method for measuring proximal and distal tibial torsion

**Fig. 1:**
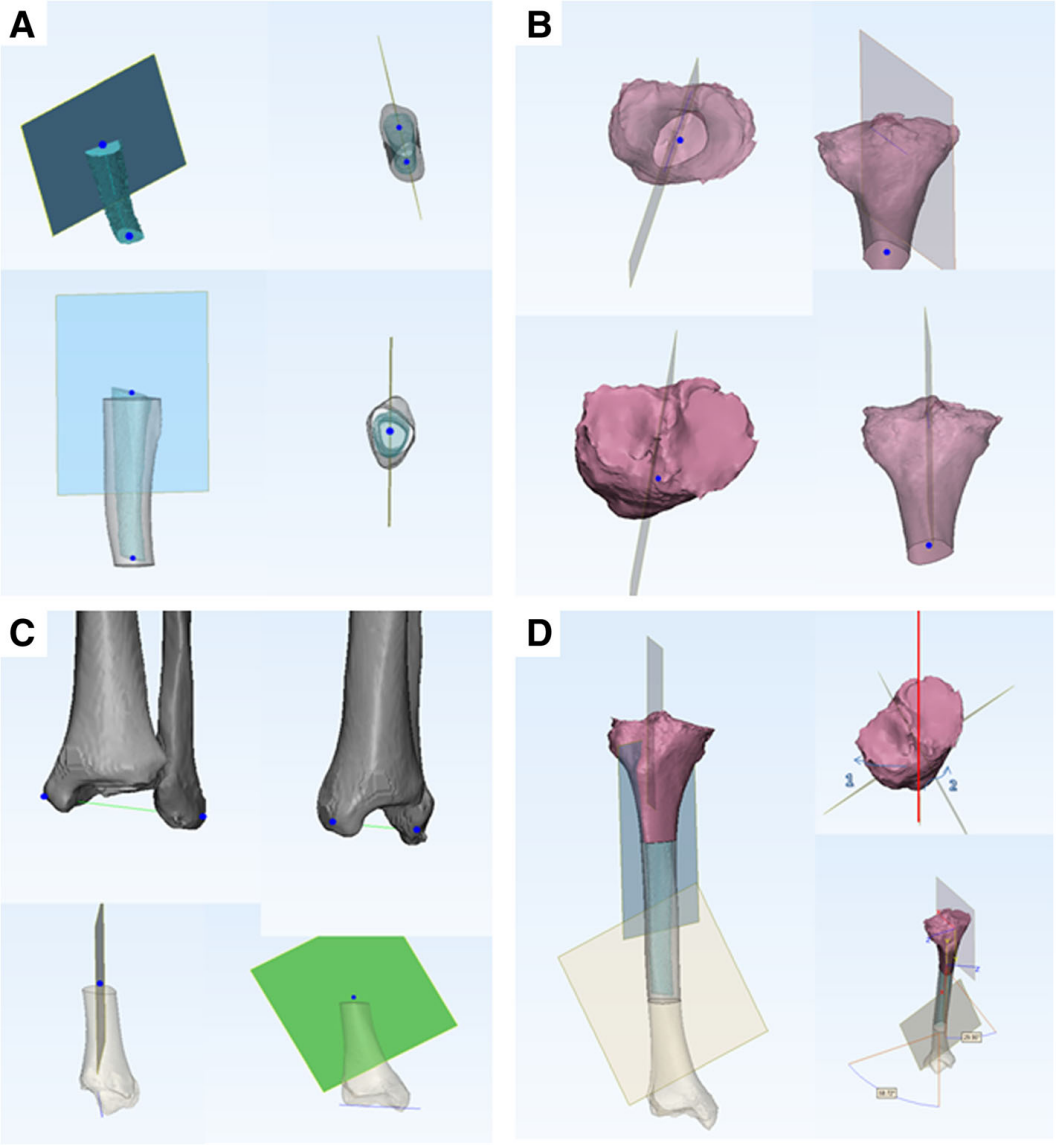
**a** Determination of the datum sagittal plane in the middle tibia portion; **b** Determination of torsion sagittal plane in the proximal tibia portion; **c** Determination of torsion sagittal plane of the distal tibia portion; **d** Form the three tibias and their respective torsion plane

Using a 3D tibial model constructed from high-resolution CT, the tibia was divided into three equal parts between the level below the osteophytes and the ankle. Using Mimics software (Materialise NV, Leuven, Belgium), a medullary cavity model of the middle tibia was constructed using high-resolution CT (layer thickness, 1.5 mm) of the lower limbs. In addition, the medullary cavity on each scanned layer fitted for a circle, with the circle centre at the midpoint of the medullary cavity of each layer. The centres of all layers were marked and an arc was generated by the Mimics software through all centre points; only one circle could be fitted. The sagittal plane of the middle tibial datum, termed the “datum plane,” was defined as the plane that fitted the created circle by covering most of the midpoints of the medullary cavity in each CT layer (Fig. 1a).

The sagittal plane of the proximal tibial torsion was determined from the line between one-third of the tibial tubercle and the deepest point of the posterior tibial end of the posterior cruciate ligament; this line was connected with the proximal one-third of the central point of the medullary cavity plane at the proximal tibia after removing any interference from the osteophytes (Fig. 1b).

The sagittal plane of the distal tibial torsion was determined according to the distal tibial torsion line on the distal articular surface of the tibia that connects the medial malleolus tip to the mid-point of the lateral border (fibular sulcus) [11, 12]. The sagittal plane was combined with the distal one-third of the central point of the medullary cavity plane at the distal tibia forming the three parts of tibia and their respective torsion plane (Fig. 1c).

Based on the torsion datum sagittal plane, termed as the “sagittal plane”, the plane coordinate system perpendicular to the sagittal plane was created, in which the coordinate plane consistent with the tibial platform direction was the axial plane. We then established a coordinate system: if the sagittal torsional plane of the distal tibia (Fig. 1d) has an acute angle in the axial direction with the datum plane and it deviates laterally in the coronal position, it is defined as the proximal tibia rotation angle for the new method. Likewise, if the torsional sagittal plane of the distal tibia (Fig. 1d) has an acute angle in the axial direction with the datum plane and it deviates to the medial side in the coronal position, it is defined as the distal tibia rotation angle for the new method.

### Traditional method for measuring proximal and distal tibial torsion and comparisons of tibial torsions measured between the new and traditional methods

Traditional tibial torsion measurements were made according to the method suggested by Mochizuki et al. [13]. Traditional tibial torsion measurements were made according to the method by Mochizuki et al., which is the angle between the line connecting the medial end with the lateral end on the proximal tibial joint surface and the line connecting the tip of the medial malleolus of the ankle joint with the tip of the lateral malleolus of the fibula.

The proximal and distal tibial torsions were compared between the new and traditional methods in each group.

#### Comparisons of alignment deviation between extramedullary and intramedullary simulated osteotomy

**Fig. 2:**
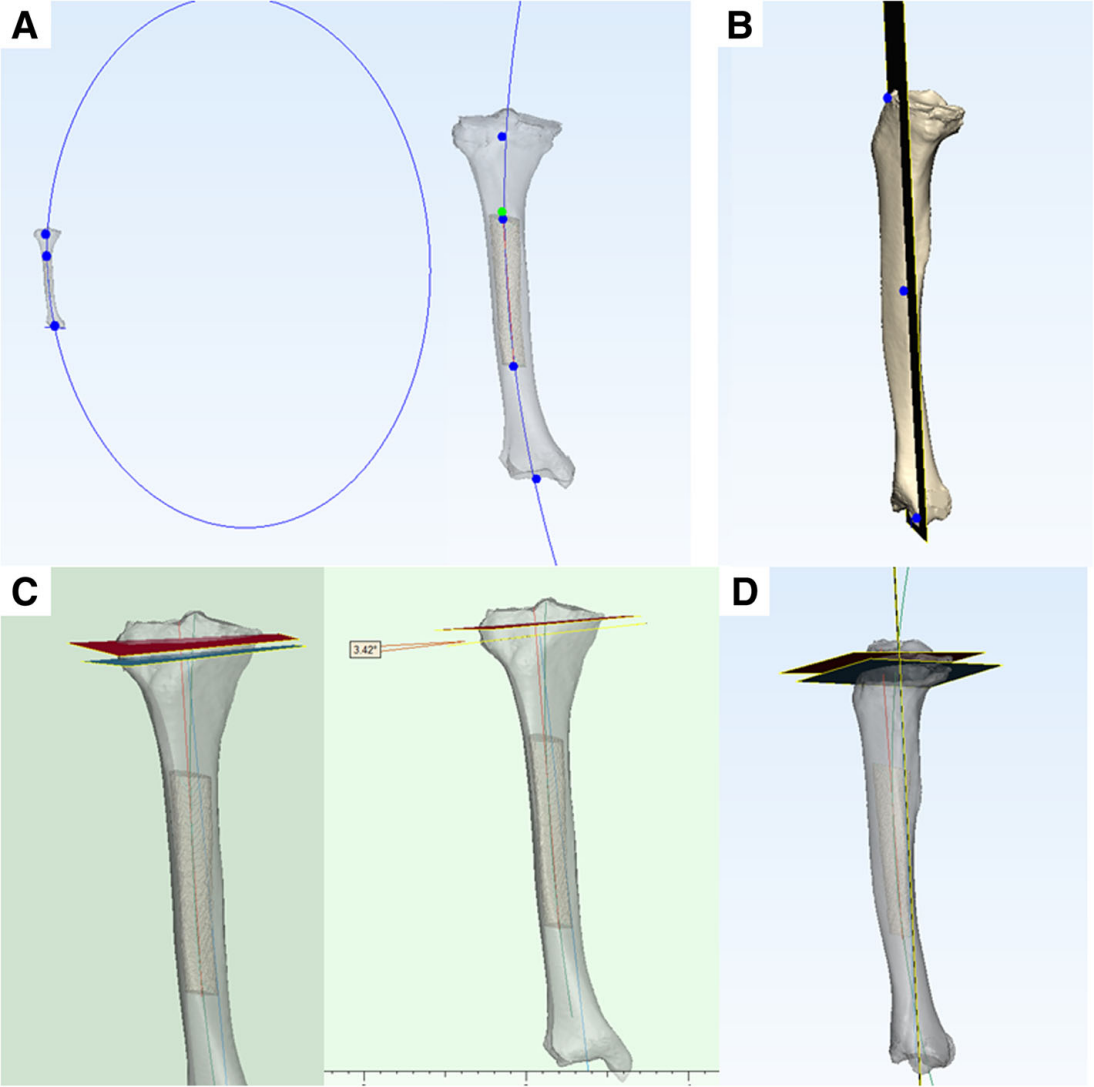
**a** The centers of medullary cavity from all layers are marked and all center points are automatically fitted into an arc, as shown the blue circle in **a** and the green arc-part of the circle in **c** and **d**. Because of its small radius, it can be approximately fitted into a straight line, as shown the red line in **a**, **c** and **d** representing the alignment of the medullary cavity. **b** Establish the coronal plane which simulates the coronal field of vision shown during surgery. **c** and **d** The blue line is the alignment line which can reflect the coronal extramedullary system alignment, and the osteotomy plane as shown the blue section plane in **c**; the red line in **a**, **c** and **d** representing the alignment of the medullary cavity, and the osteotomy plane perpendicular to the alignment as shown the red section plane in **c**

The traditional extramedullary system osteotomy method was simulated. A line was created between one-third of the tibial tubercle and the deepest point of the posterior tibial end of the posterior cruciate ligament on the 3D model of the tibia; this method was similar to that reported by Cinotti et al. [6]. A circle was made in the ankle mortise plane of the CT image in the axial plane. The circle centre was the point of distal tibial alignment. We then established an alignment line with the centre point of the line between one-third of the tibial tubercle, the deepest point of the posterior tibial end of the posterior cruciate ligament, and the point of distal tibial alignment; this force line could reflect the coronal extramedullary system alignment (Fig. 2c).

**Fig. 3:**
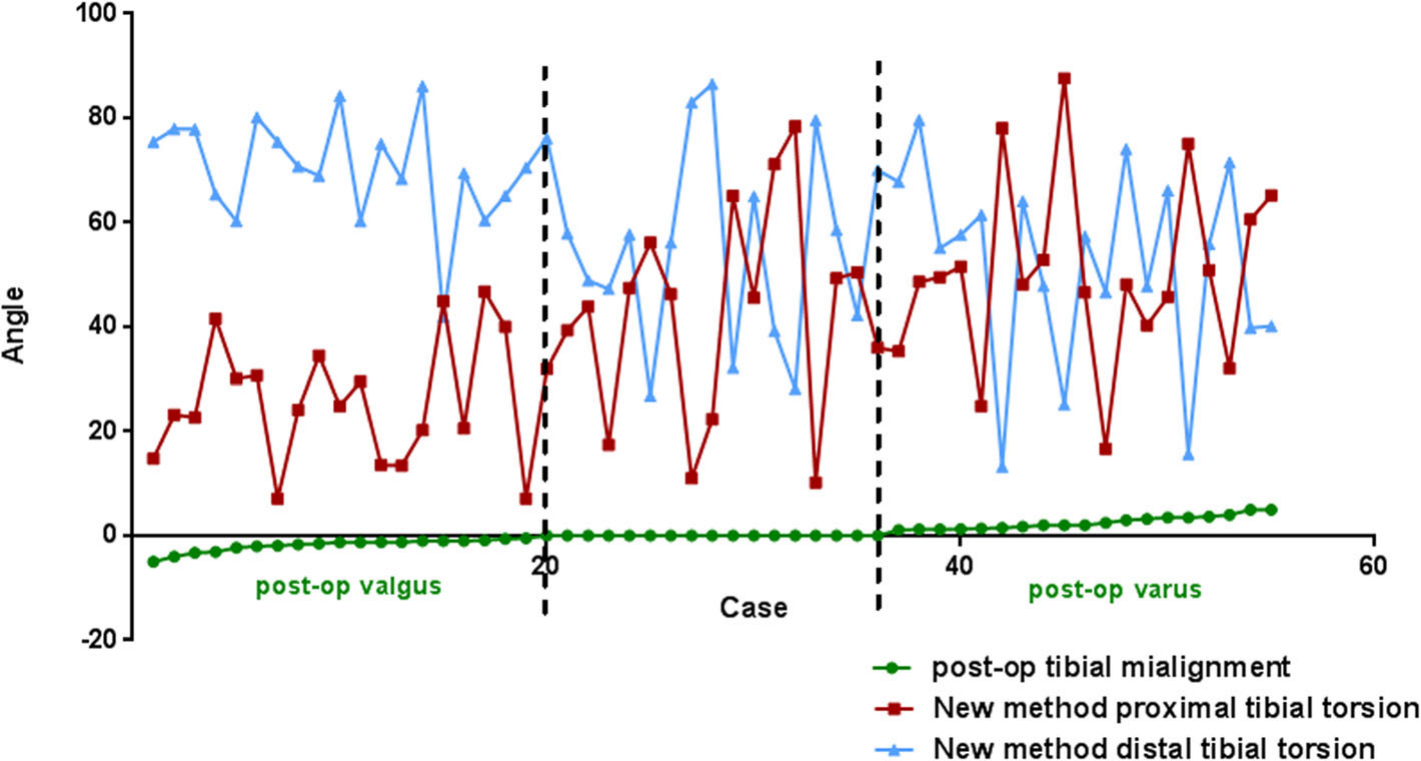
The torsion angle of the proximal tibia in the valgus group measured by the new method was smaller than that of the neutral group and tends to have statistically difference. The distal torsion of the valgus group was higher than that of normal group and varus group and tends to have statistically difference

The model for the middle tibial medullary cavity was constructed by high-resolution CT (layer thickness, 1.5 mm) for the lower limbs, and the circle was fitted with the medullary cavity as the centre on each slice of the CT scan. The circle centre was the midpoint of the medullary cavity. The centres of all layers were marked and all centre points were automatically fitted into an arc by the Mimics software. The arc passed through most of the centre points. Due to the large radius of the arc, it could be fitted to an approximately straight line, which is defined as the alignment of the medullary cavity (Fig. 2a). The cross-section was perpendicular to the alignment, representing the new middle tibial medullary cavity centre-line osteotomy method (Fig. 2c, d).

We then connected the line between the most protruding part of the lateral side of the distal fibula at the lateral malleolus, the most protruding part of medial side of the distal tibia at the medial malleolus (simulating an extramedullary system holding ankle device), and the central point of the middle medullary cavity of the tibia to establish a coronal plane, which simulated the coronal field of vision shown during surgery (Fig. 2b). The angles of the two osteotomy section planes projected on this coronal plane were compared (Fig. 2c). The valgus and angles were recorded as a negative and positive values, respectively. Comparisons with the alignment deviation measured from the post-operative radiographs, we can have the alignment improvement status by using the middle tibial medullary cavity centre-line intramedullary system osteotomy.

### Statistical analysis

The Shapiro-Wilk test was used to determine the normality of the data. Data with Gaussian distributions were compared using a one-way analysis of variance. Data with non-Gaussian distributions were compared using the Kruskal-Wallis H test. Categorical variables were analysed with the chi-squared testor Fisher’s exact test. A *p* value of < 0.05 was considered statistically significant. All statistical analyses were performed using SPSS Statistics version 22(IBM Corporation, Armonk, NY, USA).

## Results

The 55 patients were divided into three groups: varus, valgus, and neutral groups according to the postoperative alignment. There was no significant difference in the baseline characteristics among the three groups (Table 1).

**Table 1:** Comparisons of baseline between the three groups

	Post-op varus	Post-op neutral	Post-op valgus	*p* value
Gender (male/female)	*0/14*	0/10	3/11	1.000
Age (years)	65 ± 7.0	65 ± 7.0	65 ± 7.0	0.76
Height (cm)	160 ± 6.69	160 ± 6.69	160 ± 6.69	0.904
Weight (kg)	65 ± 9.05	65 ± 9.03	65 ± 9.01	0.631
BMI (kg/cm^2^)	26 ± 3.7	26 ± 3.61	26 ± 4.2	0.969
Tibia length (cm)	320.12 ± 32.02	340.86 ± 59.57	322 ± 48.72	0.691
Femur length (cm)	412.18 ± 44.17	421.98 ± 83.16	409 ± 73.41	0.820
Pre-op varus deformity (°)	8 ± 6.5	7.5 ± 6.7	8 ± 6.0	0.701

Continuous data were presented as mean ± SD. A significant difference between groups was considered for *p* < 0.05

The results of proximal and distal torsion measured by both the new and traditional methods are shown in Table 2. There were significant differences among the three groups with regard to both proximal and distal torsion (*p* = 0.03 and *p* = 0.02, respectively). As measured according to the new methods, the proximal tibial torsion of the valgus group was significantly lower than both the neutral group (*p* = 0.009) and the varus group (*p* = 0.001). The distal torsion of the valgus group tended to be higher than that of the neutral group (*p* = 0.091) and was significantly higher than that of the varus group (*p* = 0.013). There was no significant differences in either the proximal or distal torsion between the varus and neutral groups (*p* = 0.356 and *p* = 0.987, respectively). For measuring proximal and distal torsion, no significant differences were found among the three groups when using the traditional method (*p* = 0.782 and *p* = 0.753, respectively) (Table 2, Fig. 3).

**Table 2:** proximal/distal tibial outcomes evaluated by new tibial torsion method and traditional tibial method

		Post-op varus	Post-op neutral	Post-op valgus	*P* value
New tibial torsion method	Proximal	43.03 ± 21.13	41.6 ± 17.28	25.12 ± 15.22	*p* = 0.03
					Varus vs. neutral *P* = 0.356
					Valgus vs. neutral *P* = 0.009
					Varus vs. valgus *P* = 0.001
	Distal	59.61 ± 19.15	56.94 ± 20.1	75.55 ± 12.0	*p* = 0.02
					Varus vs. neutral *P* = 0.987
					Valgus vs. neutral *P* = 0.091
					Varus vs. valgus *P* = 0.013
Conventional tibial method	Proximal	26.94 ± 9.01	28.67 ± 8.01	27.67 ± 8.9	*p* = 0.782
					Varus vs. neutral *P* = 0.485
					Valgus vs. neutral *P* = 0.512
					Varus vs. valgus *P* = 0.824
	Distal	2.89 ± 2.1	3.17 ± 2.5	3.81 ± 2.3	*p* = 0.753
					Varus vs. neutral *P* = 0.501
					Valgus vs. neutral *P* = 0.702
					Varus vs. valgus *P* = 0.782

Data were presented as average mean ± SD. A significant difference between groups was considered for *p* < 0.05

**Fig. 4:**
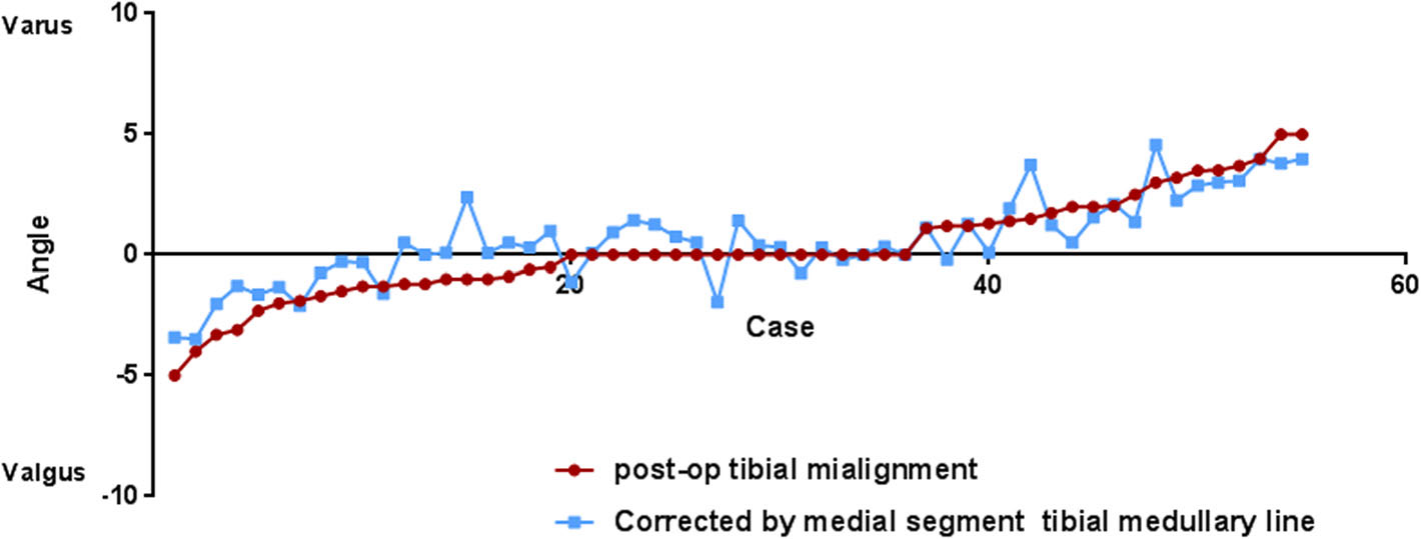
For the postoperative valgus group, the postoperative prosthesis placement deviation angle of the alignment by the traditional extramedullary positioning system method were improved by reference to the simulated osteotomy of midtibial medullary cavity alignment deviation angle. For the postoperative varus group, improvement was not obvious

In the valgus group, simulated osteotomy that was guided by the intramedullary positioning method obtained more neutral alignment compared with that guided by the extramedullary system. However, in the varus group, the improvement was not significant (Table 3; individual data presented in Fig. 4).

**Table 3:** Intragroup comparison of changes in the post-op varus and valgus groups after simulated osteotomy

Postoperative Alignment deviation(°)			
	Traditional extramedullary method	Simulated. intramedullary method	*p* value
Postoperative varus group	2.7 ± 1.3	2.4 ± 1.4	*p* = 0.597
Postoperative valgus group	−1.7 ± 1.3	−0.3 ± 1.48	*p* = 0.039

Data were presented as mean ± SD. A significant difference between groups was considered for *p* < 0.05

## Discussion

In this study, we established a new method for evaluating proximal and distal tibial torsion. Due to the different test subjects of the two methods, the tibial rotation measured by the new method is the angle between the proximal and distal torsion planes, while the tibial rotation measured by the traditional method is the angle between the proximal and distal torsion lines. The two are quite different both in methods and in values. So the two cannot be compared together. But we found some relationship between them. It is not possible to directly compare the data obtained by the two test methods. Only the correlation between the torsion and the postoperative alignment in the new method can be evaluated. Since tibial torsion showed different directions in both the proximal and distal regions, the traditional method that evaluates proximal and distal torsion together cannot completely reflect the true tibial torsion. Although Mochizuki et al. [13] studied tibial torsion separately, they did not compare postoperative alignment. Thus, the effect of tibial torsion on postoperative alignment was unclear.

Most published studies have defined the tibial torsion line using the coronal position on CT in a 2D manner [14–16]; we used 3D techniques to analyse tibial torsion. The results of this study indicated that a large tibial torsion might associated with tibial alignment. This is consistent with the findings of Takahashi et al. [8]; however, they focused on the association between tibial and fibular alignment in the sagittal position and the torsion measurement was based on the tibial medial-lateral axis in the axial plane of the CT. During surgery, tibial torsion is usually applied along the anteroposterior torsion line (i.e., the line connecting the medial one-third of the tibial tubercle to the posterior cruciate ligament in the axial plane). We used the axial torsion line for localization in the same manner as we use in surgery; therefore, our results are much closer to those achieved in clinical practice. The method used by Cinotti et al. [6] for determining proximal tibial torsion intraoperatively, which is similar to the method used in this study, uses the axial plane of a CT for analysis. To avoid a tibial cut toward a varus angle, the extramedullary alignment system should be translated medially by approximately 9–11 mm. However, Cinotti et al. did not use a 3D method to simulate osteotomy and verify their conclusions; in addition, they did not link the findings with postoperative outcomes.

Akagi et al. [7] also measured the axial anteroposterior torsion line and suggested that the medial edge of the tibial tubercle and the insertion of the posterior cruciate ligament can better reflect tibial torsion. However, their study was based on an axial view from CT, which does not intuitively simulate surgery. Although Gonzalez-Carbonell et al. [17] used 3D technology to simulate the tibial model, their study focused on the distribution of tibial force by torsion and did not include clinical results. In the present study, we established a 3D model of the tibia and strictly simulated extramedullary system osteotomy, which is consistent with surgery. Simultaneously, the computer automatically obtained the simulated alignment of osteotomy centre line of the mid-tibia marrow cavity, which is more accurate than the manual extramedullary system procedure used for measuring alignment.

Our results indicate that obtaining tibial alignment through the mid-tibial medullary cavity centre-line could effectively correct deviations found in the post-op valgus group. In addition, Simmons et al. [10] showed that intramedullary localization could produce an accurate tibial alignment in patients with varus knee, which could lead to better outcomes than those achieved by the traditional extramedullary system. This finding is consistent with our results from the simulated osteotomies. Therefore, we recommend that when internal rotation of the proximal tibia is too small and the external rotation of the distal tibia is too large, the use of an intramedullary localization system should be considered.

Our new method is to separate the tibial torsion into 2 parts, proximal and distal. The proximal torsion part is measured more akin to clinical method surgeons used during surgery. And both measurements were based on 3D, since it’s more accurate than the traditional method we used to measure on CT or DR’s 2D images. In the previous study, traditional method has been used to evaluate tibial torsion, which is determined by transtibial axis, that’s not akin to the clinical practice. We think that’s the reason why the old method did not recognize any difference in respect to the post-operative alignment.

Our study has several limitations. First, the sample size was small. However, the torsion angles evaluated widely varied to present some knees with large tibial torsion. Second, our study was performed at a single centre; future studies should be extended to include multi-centre data. Third, our conclusions were only based on simulations and further clinical validation using intramedullary localization for analysing patients with possible misplaced alignment is needed.

Last but not least, the case which we found have a higher torsion by new method preoperatively, also observed a large torsion intraoperatively, since our new method used the same method to decide proximal torsion as we used in TKA. But we couldn’t quantify the validation during surgery. This new method has not been validated and need to be validated before using it routinely during surgery.

## Conclusions

According to the new tibial-rotation evaluation method, knees that had a small proximal tibial and a large distal external torsion tended to have valgus deviation after knee arthroplasty. The use of an intramedullary system may help reduce this deviation.

## Data Availability

Not applicable. All the data is already reflected in the manuscript.

## Abbreviations

3D: 3-dimensional
CT: Computed tomography
TKA: Total knee arthroplasty
